# Differentiated service delivery models for antiretroviral treatment refills in Northern Nigeria: Experiences of people living with HIV and health care providers–A qualitative study

**DOI:** 10.1371/journal.pone.0287862

**Published:** 2023-07-10

**Authors:** Bazghina-werq Semo, Nnenna Ezeokafor, Sylvester Adeyemi, Zipporah Kpamor, Cyrus Mugo

**Affiliations:** 1 Global Health Division, Chemonics International, Washington DC, United States of America; 2 Maryland Global Initiative Cooperation, University of Maryland, Abuja, Nigeria; 3 Global Health Division, Chemonics International, Abuja, Nigeria; 4 Department of Research and Programs, Kenyatta National Hospital, Nairobi, Kenya; Family Health International: FHI 360, UNITED STATES

## Abstract

Differentiated service delivery (DSD) and multi-month dispensing (MMD) of antiretroviral therapy (ART) have improved treatment adherence and viral suppression among people living with HIV (PLHIV), and service delivery efficiency. We assessed the experiences of PLHIV and providers with DSD and MMD in Northern Nigeria. We conducted in-depth interviews (IDI) with 40 PLHIV and 6 focus group discussions (FGD) with 39 health care providers across 5 states, exploring their experiences with 6 DSD models. Qualitative data were analyzed using NVivo®1.6.1. Most PLHIV and providers found the models acceptable and expressed satisfaction with service delivery. The DSD model preference of PLHIV was influenced by convenience, stigma, trust, and cost of care. Both PLHIV and providers indicated improvements in adherence and viral suppression; they also raised concerns about quality of care within community-based models. PLHIV and provider experiences suggest that DSD and MMD have the potential to improve patient retention rates and service delivery efficiency.

## Introduction

Globally, there are 38 million people living with HIV (PLHIV), with over 28 million currently receiving lifelong antiretroviral therapy (ART) [1, 2]. Although national HIV programs in resource-limited settings have been successful in enrolling PLHIV on ART, these efforts have not been supported by a corresponding expansion in health resources, leading to congestion in clinics and long waiting times for patients as a result of overburdened health facilities [3]. In line with World Health Organization (WHO) recommendations, many countries have gradually adopted multi-month dispensing (MMD) (3–6 months) of ART for clinically stable patients who are responding well to treatment [4, 5].

In addition to MMD, several countries with high burdens of HIV have also adopted patient-centered differentiated service delivery (DSD) models for ART drug refills designed to bring services closer to people (in their homes or communities) and to reduce the burden on health facilities [4]. Common ART drug refill models include: appointment spacing and fast-track drug refill [6–8]; patient- and provider-led community ART refill groups [9–11]; home-based ART delivery [2, 9, 12–14]; refills through private pharmacies [2]; and community ART distribution centers [6, 15, 16].

Compared to standard facility-based care, DSD models lead to similar treatment outcomes, as measured through viral suppression and rates of retention in care [9]. This has been demonstrated in multiple countries in sub-Saharan Africa, including Nigeria, South Africa, Malawi, Mozambique and Uganda [3, 6, 13, 17]. In Mozambique, community ART refill groups improved retention rates from 52% to 91.8% at 48 months [6, 18]. DSD models have also led to improvements in quality and efficiency of service delivery [9, 19–22]. In South Africa, patients receiving MMD at 6-month intervals reported saving time and money due to shorter waiting times and the need for fewer visits to clinics [2, 17, 23, 24]. In light of health care worker shortages, DSD models have also given providers the opportunity to focus on PLHIV with advanced disease [17, 25–27].

Nigeria has an estimated 1.9 million PLHIV, with approximately 1.4 million currently on ART [28]. Prior to the COVID-19 pandemic, the majority of PLHIV in Nigeria received monthly ART refills from health facilities, leading to long waiting times and clinic congestion [29, 30]. In March 2020, COVID-19 movement restrictions necessitated a major change in the delivery of health services for PLHIV, to mitigate treatment interruptions. Thus in Nigeria, COVID-19 became a major driver for the increased uptake of MMD and various DSD models [31]. The effects of this transition in Nigeria, and the resulting experiences of PLHIV and providers with MMD and DSD models of care, have not been studied.

The Strategic HIV/AIDS Response Program Task Orders (SHARP TO1 and TO3), 2-year projects funded by the U.S. Agency for International Development (USAID) and implemented by Chemonics International, support HIV care and treatment services in 11 states and approximately 200 facilities in Northern Nigeria. The HIV prevalence in Northern Nigeria ranges between 0.2–2.0 percent [32, 33]. The region is characterized by insecurity leading to poor access to treatment, and religious and social norms that affect healthcare utilization [34, 35]. Over 170,000 PLHIV have received lifelong ART through these projects. We conducted this qualitative study to understand the experiences of PLHIV and ART providers following the implementation of MMD and various DSD models of care.

## Materials and methods

### Study design and population

This qualitative study was part of a larger mixed-methods study conducted in Northern Nigeria, between April 2021 and March 2022, on the implementation of DSD models of care across 5 states in Northern Nigeria (Adamawa, Bauchi, Borno, Kano and Niger). We identified 16 facilities offering at least 1 of 6 DSD ART refill models (community pharmacy; patient-led community ART refill groups; provider-led community ART refill groups; decentralized ART refill centers; family ART refill groups; and standard of care facility-based ART refill) to participate in the study. Study sites were selected through purposive sampling informed by a survey completed by heads of facilities in the 11 states in Northern Nigeria in which the SHARP TO1 and TO3 projects operate. The facilities (all hospitals) were located in Adamawa (4), Kano (4), Niger (3), Bauchi (3) and Borno (2) states. We selected hospitals located in urban settings bearing in mind the security situation in Northern Nigeria.

### PLHIV interviews

We conducted 40 in-depth interviews (IDIs) with PLHIV aged 18 years and older who had been in care for 2 or more years and reported receiving ART through any 1 of the 6 DSD models. IDIs were conducted with PLHIV from health facilities located in Adamawa (3), Bauchi (1), Kano (3) and Niger (2) states. For each of the 6 DSD models, we estimated 6–9 patients would be adequate to provide diverse information regarding their experiences receiving care through a specific model to reach saturation.

Clinic staff assessed patient eligibility during routine clinic visits through self-report and medical records. We conducted the IDIs in person using a semi-structured questionnaire.

### Provider focus group discussions

We conducted 6 focus group discussions (FGDs) with health care providers recruited from 6 of the 16 selected facilities. FGDs were conducted with providers in health facilities located in Adamawa (1), Bauchi (1), Borno (2), Kano (1) and Niger (1) states. We selected facilities that offered diverse DSD models, ensuring each of the 6 models was represented in at least 2 of the FGDs. Nominated by clinic managers, the providers (doctors, clinical officers, nurses, counselors, pharmacists/pharmacy technologists, laboratory technologists and other health workers) were 18 years or older and had at least 1 year of experience delivering services in HIV clinics. We conducted FGDs in person using a structured guide with the participation of 10 providers in each group. FGDs were conducted among a mix of providers working in a particular facility.

All interviews were conducted by trained interviewers solely recruited for the purpose of the study.

### Conceptual model

**Fig 1:**
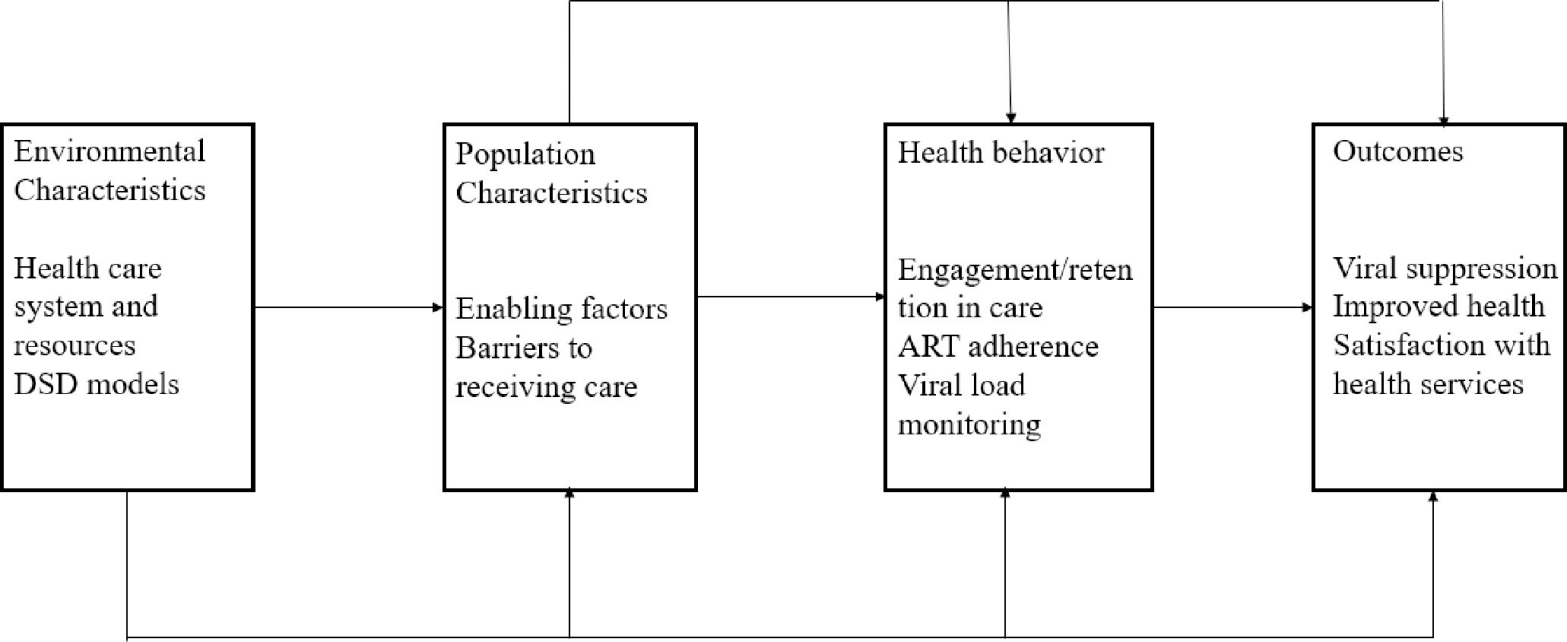
Adaptation of the Andersen behavioral model for health care utilization in Northern Nigeria.

We developed the IDI and FGD guides using components of the Andersen Behavioral Model for Health Care Utilization [36] to gather information on the following areas: acceptability of and preferences for DSD models; satisfaction with care; and the impact of DSD models on ART adherence, engagement in care, viral load monitoring, and cost of care (Fig 1).

### Data collection

IDIs and FGDs were conducted in English between August-December 2021, audio-recorded, and transcribed. Most patients were proficient in English, while some responded in English and Hausa (local language). During transcription, Hausa words were translated into English. Demographic data were captured for each participant using Open Data Kit (ODK) and were exported and analyzed in MS-Excel (2019).

### Data analysis

We used a deductive thematic analysis approach to identify parent and subthemes, creating a start list of codes from components of the Andersen Behavioral Model. IDI and FGD transcripts were reviewed and analyzed by primary analyst (NAE) and independent analysts (SA and ZK). The analysts jointly compared, discussed, and modified the applied themes and subthemes, and added additional themes accordingly to the codebook, resolving any disagreements through discussions. Qualitative data were analyzed using NVivo^®^1.6.1. Demographic data were summarized in MS-excel using counts, proportions, medians, and interquartile ranges (IQR).

### Ethics approval

We received Institutional Review Board (IRB) approvals from the National Health Research Ethics Committee of Nigeria (NHREC Approval Number NHREC/01/01/2007-28/06/2021) and the University of Maryland Baltimore Institutional Review Board (HP-00097414). PLHIV provided informed written consent, while health care workers provided documented verbal consent prior to enrolment in the study.

## Results

### Participant characteristics

IDI participants were PLHIV with a median age of 39 years (IQR 24–43). Of the 40 participants, 22 (55%) were male, 23 (58%) were currently married or co-habiting, and 25 (63%) were accessing care at secondary facilities (e.g., district hospitals). The participants had received HIV care and treatment services for a mean duration of 9 years. Health care providers participating in FGDs had a median age of 39 years (IQR 30–45). Male and female providers were equally represented and consisted primarily of clinical officers (20%), nurses (13%) and counselors (13%). See Table 1 for detailed characteristics.

**Table 1:** Characteristics of FGD and IDI participants.

Characteristics	Patient, N = 40	Health care Providers, N = 39
	*Median (IQR)/n (%)*	*Median (IQR)/n (%)*
**Age**	39 (24–43)	39 (30–35)
**Sex**		
Female	18(45)	19(49)
Male	22(55)	20(51)
**Duration on ART (years)**	9 (5–13)	
**Marital Status**		
Single (never married)	12(30)	
Married/co-habiting	23(57.5)	
Widowed	3(7.5)	
Divorced/separated	2(5.0)	
**Employment Status **		
Employed, Professional	9(23)	
Self-employed, Professional	15(38)	
Casual	4(9)	
Unemployed	12(30)	
**Education**		
Primary class	6(15)	
Secondary	12(30)	4(10)
Polytechnic	16(40)	11(28)
University/College	6(15)	24(62)
**Facility Type**		
Tertiary	15(37.5)	
Secondary	25(62.5)	
**Health Provider**		
Doctors		2(5)
Clinical officers		8(20)
Nurses		5(13)
Pharmacists/ pharmacy technologists		3(8)
Counselors		5(13)
Laboratory technologists		3(8)
Others		13(33)

Empty boxes denote variable is not applicable to the target population

### Theme 1: Acceptability of DSD models

Patients and providers found DSD models acceptable due to factors such as reduced frequency of clinic visits and increased convenience of services. One patient recommended: “What I really like is how[I] am collecting my drugs for 6 months, I really like how drugs are given to patients for six (6) months…but I want it to be extended for one year.” (43-year-old female patient receiving treatment in a community pharmacy in Adamawa State).

Another patient found the model acceptable due to its convenience and user-friendliness: “One of the major differences is that now I can even sit in my house and call and someone from the clinic will bring the medicine for me at home. Once we agree on the date, they will bring [ART] to the house and even enter for us to exchange pleasantries.” (40-year-old male patient receiving treatment through a provider-led community group in Bauchi State).

Providers reported improved convenience for patients: “We [Health Care Workers] also moved ahead to dispensing six months which the patient just comes twice a year, and which is less stressful.” (Provider in Niger State).

Providers also reported improved convenience for themselves: “It [DSD model] allows us [providers] even the staff here to do some other things [because] whatever thing we are going to do here is for their [patient] benefit so it is of a great advantage.” (Provider in Niger State).

### Theme 2: Preferences for DSD models

The preferences of PLHIV for various DSD models were influenced by factors such as convenience, stigma, trust, and cost of care.

#### Convenience

Some PLHIV indicated that they preferred community pharmacies because their close proximity to home, daily operation, and long hours made collection of ART convenient.

According to a patient: “I think I prefer that community pharmacy because I go there at my own convenience whether in the morning, afternoon or night; any time I have time but within the date they gave me I will go there and collect my drugs.” (43-year-old female patient receiving drugs in a community pharmacy in Kano State).

Another patient remarked: “I don’t need to start paying [for] transport to the clinic to get it [ART] and this [community pharmacy] is just very close to my house so I just walk down and walk back so I would still prefer the community pharmacy.” (24-year-old male patient receiving ART in a community pharmacy in Niger State).

#### Stigma

Some PLHIV indicated a preference for the home delivery of ART model (a subcomponent of provider-led community groups) to alleviate potential stigma and discrimination. One patient noted: “I will prefer home delivery; home delivery will be better because at least stigmatization and people get to know that you are a carrier [HIV positive] or things like that so I think that will help.” (24-year-old male patient in a provider-led community model in Niger State).

Conversely, some providers identified facility-based delivery of ART as a model that reduced stigma. One provider, for example, noted that patient familiarity with providers facilitated service delivery by alleviating their concerns about stigma: “I think for me based on how the clients are behaving towards DSD models, I feel it’s just better to keep them in the facility because most of them are still not open to the DSD like I said earlier because of the whole secrecy and all. They still prefer to come to the facility [to meet] people that they are used to, people they see every day because the program does not change staff all the time so at least a case manager [Counselor] could even be having [managing] a client that has taken his/her refills since 2015 so you see this gives them more confidence and then they feel that their situations are confidential because it is one particular person I [they] see all the time.” (Provider in Borno State).

#### Trust

Some PLHIV indicated that they felt safer and better cared for at health facilities and therefore preferred the facility-based ART refill model (standard of care). One patient commented: “I prefer coming to the hospital to collect my drugs, and I have not collected my drug in the pharmacy or any other place apart from the hospital. It is better and safer for me.” (29-year-old male patient in a standard of care model in Kano State).

Another patient mentioned: “I don’t want anyone to collect my drugs, because I want to always see the doctor, so that I will explain how I am feeling to the doctor so that he will know me very well. I don’t have experience collecting refills in community groups because I don’t like anyone to collect my drugs. “(48-year-old female using facility-based ART refill model in Adamawa State).

#### Cost of care

Some PLHIV indicated a preference for the free of charge standard of care model over the fee-based community pharmacy model. One patient mentioned: “You must be able to pay the pharmacy some certain amount [1000 naira] for every 6 months you visit there, but in [the facility] IDH you don’t pay any money, it is free of charge.” (43-year-old female patient currently accessing drugs in a community pharmacy in Kano State).

### Theme 3: Satisfaction with DSD models

Both PLHIV and providers expressed a high degree of satisfaction with the introduction of DSD models. Specifically, patients enjoyed the fast-track services at health facilities, while providers were pleased with the reduced levels of facility congestion. When asked about how much time is typically spent at clinic visits, a patient remarked: “I spend 30 minutes now seeing my doctor, and it’s appropriate, but people with different problems spend more than one hour seeing the doctor. I think it is appropriate and as for me I prefer it shorter.” (38-year-old female patient accessing treatment through the facility-based ART refill model in Adamawa State).

Another patient stated: “Before COVID-19, there was congestion with a large number of clients at the facility; we normally spent 10 hours waiting at the waiting room before collecting our drugs. After COVID-19 we spend fewer hours at the facility because they have distributed clients to various DSD model”. (38-year-old female patient accessing ART through the facility-based ART refill model in Adamawa State).

Similarly, providers expressed satisfaction with the lower levels of congestion at the clinics brought about by the introduction of the DSD models. When asked about the service delivery modifications and their appropriateness for patients and staff, a provider responded: “Well, it prevented, or it stopped patient waiting time cause usually in a day we might see up to 70 patients, 60 even as more as 80 but with the six-month dispensing it reduces to 30, 40, 20 so it gives us enough time to do other things too.” (Provider in Niger State).

Another provider noted: “The modification is appropriate and its makes work easier for both staff and clients, we don’t have to track our clients now, we don’t have huge number of defaulters now, because we take services to their location, its makes work easy for us because we don’t have number of defaulters.” (Provider in Adamawa State).

Some providers, however, cautioned that guidelines were needed to support and standardize the implementation of DSD. One provider mentioned: “We need provision of SOPs and guidelines to actualize the implementation of the MMD and DSD Models.” (Provider in Adamawa State).

### Theme 4: Perceived quality of care

Despite finding DSD models acceptable, some patients expressed concern about the quality of care they received in health facilities after the implementation of the new models. According to a patient: “The thing I will want them to change is this, before COVID came they use to give us health talk when we come in but now, they don’t. Briefly do health talk, weigh us, do adherence and then we pick up our refills in good time.” (43-year-old female patient accessing treatment through the facility-based ART refill model in Bauchi State).

Similarly, another PLHIV said: “Before the COVID, services were being offered normally that is a patient will be weighed, health talk will be made, and other things but since COVID came, no health talk, no full attention was given to us to avoid congestion and maintenance of social distance, the thing I will want them to change is this, I want them to include [health talk and vital signs check] so that new client will have more knowledge about the disease condition.” (22-year-old female patient in the family group model in Bauchi State).

Some providers expressed concern that the longer refill intervals and community ART dispensing models could negatively impact quality of care and overall health outcomes. One believed that patient health conditions would worsen without regular clinic visits: “If they have another health issues, they will not come to the facility because they have enough drugs to take at home.” (Provider in Adamawa State).

Another provider stated: “There is visible and significant improvement amongst the clients that pick up their drugs from the hospital than in the clients that collects their medications outside.” (Provider in Kano State).

### Theme 5: Impact of DSD models

The majority of PLHIV indicated that the DSD models and MMD strategies improved adherence to ART, retention in care and viral suppression, and reduced cost of care. Providers, on the other hand, reported mixed feedback regarding the impact of DSD models on adherence.

#### Adherence and engagement in care and viral suppression

Many PLHIV reported that the DSD models and MMD supported their adherence to ART. One patient noted: “The duration (two visits per year) affects my adherence [positively] as well as my ability to attend clinics.” (A 38-year-old female patient receiving treatment through the facility-based ART refill model in Adamawa State)

Some providers shared similar views: “It has impacted greatly to the client’s attendance as well adherence to treatment, receiving refills and viral load suppression.” (Provider in Adamawa State).

Providers also reported improved viral suppression among patients in various DSD models: “Well, the good thing is it [DSD] helps the clients because somehow, we have seen to an extent improvement in the viral load and viral load suppression.” (Provider in Niger State) Another provider remarked: “You have more clients that are virally suppressed compared to then [prior to DSD] in both adults and children.” (Provider in Borno State).

Several providers, however, indicated that the DSD models had negatively impacted adherence to treatment: “Some of these clients feel relax taking drugs, they forget their clinic day because they have drugs at hand.” (Counselor in Adamawa State).

One provider posited that a lack of regular contact with providers compromised patients’ abilities to adhere to their treatment:”The fact that there’s reduced contact with the counselors there’s this temptation of let me [patient] just not take [ART] today and things like that but when it was one month of course [ART refill] the more you come in contact with the adherence counselor he gives you pep talk and then you know but now it’s reduced so that thing about adherence I think is the major thing.” (Provider in Niger State).

#### Cost of care

PLHIV and providers noted that DSD models reduced cost of care for patients, especially the high transportation costs associated with travelling to health facilities for monthly visits.

*A patient commented*. “Sometimes, I don’t normally have money to come, I used to miss my appointment sometimes like two three days because of transport money.” (33-year-old female patient receiving treatment through the facility-based ART refill model in Niger State).

*Another remarked*. “It also reduces the cost of transportation because you only come twice a year.” (43-year-old male patient receiving treatment through the decentralized ART refill model in Adamawa State).

*A provider noted*. “The advantage is [that] those refills are close to the client and ideally the community refills and any other refills that is outside the facility or outside the hub site is supposed to be much easier for the clients because they do not have to spend transportation money to come to the hub site for their refills or our viral load sample collection.” (Provider in Borno State).

## Discussion

This study showed that PLHIV and health care providers perceived MMD and DSD models for ART refill to be acceptable and to be facilitators of better HIV outcomes and decongestion of health facilities. Most patients in the study reported satisfaction with 6-month ART refills, with others preferring and recommending longer intervals between medication refills. Similar findings have been noted in Ethiopia and Thailand, with patients preferring longer (up to 1 year) ART refills [26, 37].

Factors that influenced patient preferences for DSD models included: convenience; stigma; trust; and cost of care. For example, PLHIV tended to prefer community pharmacies compared to other models due to their greater proximity to home than the typical facility, and the convenience of longer hours of operation. These findings are similar to a study conducted in Kenya in which patients preferred community pharmacies over other models because of their extended hours of operation and convenient locations [38]. Similarly, PLHIV who considered facility attendance to be stigmatizing expressed a preference for community home delivery of ART. Studies in Botswana [2], Kenya [34] and Uganda [35] reported similar findings in which patient perceptions of stigma and discrimination reduced considerably with community-based home delivery of ART by health care workers. The findings from our study and those of other countries underscore the continued need to address stigma and discrimination in HIV programs.

Our findings regarding cost of care corroborate those of previous studies in the region [26, 39–43], with PLHIV on MMD and receiving treatment through community-based models reporting reduced transportation costs and other out-of-pocket expenses due to infrequent clinic visits. Patients who preferred facility-based ART refill perceived this model to provide greater anonymity, and thus safety, and to offer better quality of care. Some patients found seeing a provider at a facility reassuring. In facility-based interactions, providers gained the trust of their clients, obtained better insight into issues of patient adherence, and were well positioned to identify and address other psychosocial issues. A similar perception among some PLHIV, that facility-based care could provide some measure of protection from community stigma, was shown in a study in Uganda in which a high uptake of facility-based models was linked to patient fears of unintentional disclosure of their HIV status by peers living in the same neighborhoods [39, 44].

Furthermore, facility-based services were perceived to be more affordable. In Northern Nigeria, community pharmacies are known to charge nominal fees for ART refills that some PLHIV find prohibitive, leading them to prefer other service delivery models that are free of charge. We noticed similar findings in Northwest Ethiopia and Botswana. In Ethiopia, fees associated with drug refills led to financial concerns that had a negative impact on adherence [26]. In Botswana, the willingness of PLHIV to access their medication through private pharmacies dropped from 60.67% to 44.2% when they were expected to pay a dispensing fee [2].

More broadly, respondents reported that the introduction of DSD models led to less congestion in health clinics and reduced patient waiting times. In addition, some PLHIV identified planning for other aspects of patient care during the ART refill visit (e.g., viral load sample collection), as a positive factor. The decongestion of facilities allows providers more time to provide high quality care to fewer patients, and to focus on newly initiated or clinically unstable clients [40].

Overall, providers and PLHIV indicated that DSD models helped improve patient adherence and retention in care. However, some providers in our study and in others in the region [15] expressed fears that MMD could result in inconsistent compliance to medication. According to studies throughout the region, these fears have not been realized. In fact, studies in Nigeria [45], South Africa, Uganda, Zimbabwe and Guinea showed that a higher percentage of PLHIV on 6-monthly MMD remained in care 18 months after initiation of ART [8, 15].

Furthermore, after the expansion of DSD models for ART refills, several providers in our study noted higher levels of viral suppression among PLHIV. While other factors such as the roll out of the medication dolutegravir occurred during the same period, we can attribute the introduction of DSD models to some of the improvements in viral suppression, adherence, and engagement in care. Our findings are comparable to a multi-country analysis of 21 U.S. President’s Emergency Plan for AIDS Relief (PEPFAR) countries that showed steady increases in virologic suppression among patients accessing MMD, a fundamental characteristic of any DSD model [31].

This study had a few limitations. As we set about capturing a range of perspectives from PLHIV availing themselves of the different service delivery models operational in our setting, we found that some respondents lacked clarity about the specific DSD model they were accessing for ART refills. We resolved this challenge by instructing the interviewers to spend additional time clarifying the DSD model. Due to security issues and limited research funding, most of our respondents were mainly from urban settings in the Northeast of Nigeria. While we would not anticipate a wide variation in experiences with the various service delivery models, additional research focusing on rural populations may be required for a more complete understanding of the perspectives of PLHIV and providers outside of urban settings.

## Conclusion

PLHIV and provider experiences suggest that MMD and DSD models are acceptable and have the potential to improve patient retention, viral suppression, and efficiency in service delivery. As HIV programs continue to decentralize ART refill at the community level, it is important to continually incorporate lessons from patient and provider experiences in order to improve existing service delivery models and generate ideas for new ones.

## Supporting information

S1 File Inclusivity in global research. (DOCX)
